# The Characterization of *GSDMB* Splicing and Backsplicing Profiles Identifies Novel Isoforms and a Circular RNA That Are Dysregulated in Multiple Sclerosis

**DOI:** 10.3390/ijms18030576

**Published:** 2017-03-07

**Authors:** Giulia Cardamone, Elvezia Maria Paraboschi, Valeria Rimoldi, Stefano Duga, Giulia Soldà, Rosanna Asselta

**Affiliations:** 1 Department of Biomedical Sciences, Humanitas University, Via Manzoni 113, 20089 Rozzano, Milan, Italy; giulia.cardamone@st.hunimed.eu (G.C.); elvezia_maria.paraboschi@humanitasresearch.it (E.M.P.); valeria.rimoldi@humanitasresearch.it (V.R.); stefano.duga@hunimed.eu (S.D.); rosanna.asselta@hunimed.eu (R.A.); 2 Humanitas Clinical and Research Center, Via Manzoni 56, 20089 Rozzano, Milan, Italy

**Keywords:** *GSDMB*, alternative splicing, nonsense-mediated mRNA decay, circRNA, multiple sclerosis

## Abstract

Abnormalities in alternative splicing (AS) are emerging as recurrent features in autoimmune diseases (AIDs). In particular, a growing body of evidence suggests the existence of a pathogenic association between a generalized defect in splicing regulatory genes and multiple sclerosis (MS). Moreover, several studies have documented an unbalance in alternatively-spliced isoforms in MS patients possibly contributing to the disease etiology. In this work, using a combination of PCR-based techniques (reverse-transcription (RT)-PCR, fluorescent-competitive, real-time, and digital RT-PCR assays), we investigated the alternatively-spliced gene encoding Gasdermin B, *GSDMB*, which was repeatedly associated with susceptibility to asthma and AIDs. The in-depth characterization of *GSDMB* AS and backsplicing profiles led us to the identification of an exonic circular RNA (ecircRNA) as well as of novel *GSDMB* in-frame and out-of-frame isoforms. The non-productive splicing variants were shown to be downregulated by the nonsense-mediated mRNA decay (NMD) in human cell lines, suggesting that *GSDMB* levels are significantly modulated by NMD. Importantly, both AS isoforms and the identified ecircRNA were significantly dysregulated in peripheral blood mononuclear cells of relapsing-remitting MS patients compared to controls, further supporting the notion that aberrant RNA metabolism is a characteristic feature of the disease.

## 1. Introduction

Autoimmune diseases (AIDs) are pathologic conditions characterized by an inappropriate response of the immune system towards self-antigens. It is thought that both genetics and environmental factors play a role in the predisposition to these diseases [1]; however, the etiology of AIDs remains largely unknown. In the last years, the importance of alternative splicing (AS) in the pathogenesis of AIDs has increasingly emerged [2,3].

AS is a widespread post-transcriptional mechanism that increases the information content of the transcriptome through the expression of different mRNAs from single genes [4]. More than 95% of human genes undergo AS [5], implicating a role of this mechanism in all cellular processes. Indeed, AS concerns a vast number of genes involved in many immune system functions, such as T-cell activation and migration, cytokine response, and T-cell homeostasis and apoptosis, all being crucial to prevent the breakdown of self-tolerance and the development of autoimmunity [6,7]. It has been also suggested that AS may increase the immunogenicity of autoantigens by creating novel epitopes that might break the immune tolerance [8]. Therefore, it is not surprising that several AIDs have already been associated with an altered expression of AS isoforms, including rheumatoid arthritis, multiple sclerosis (MS), autoimmune thyroid diseases, and systemic sclerosis [9,10,11,12,13,14].

AS regulation is complex and involves both *cis*-acting elements (exonic or intronic splicing enhancers and silencers) and *trans*-acting factors (serine-arginine (SR) proteins and heterogeneous nuclear ribonucleoproteins (hnRNPs)), as well as RNA and chromatin structures [15]. Among mechanisms responsible for AS regulation, the production of circular RNAs (circRNAs) has been recently hypothesized. CircRNAs are a large class of RNAs that form covalently-closed continuous loops. Exonic circRNAs (ecircRNAs) are products of spliceosomal backsplicing, a process in which a downstream 5′ splice site is joined to an upstream 3′ splice site [16]. As the biogenesis of circRNAs competes with pre-mRNA splicing for the alternative pairing of 5′ and 3′ splice sites [17,18], backsplicing may cause either an alternative modality of splicing or the degradation of the remaining linear pre-mRNA [16]. Moreover, circRNAs may act as molecular sponges for splicing regulatory factors, thus influencing the pre-mRNA splicing, and for microRNAs [19,20].

The focus of the present work was the characterization of *GSDMB*, an alternatively-spliced gene located on the 17q12 *locus*, which was repeatedly associated with susceptibility to asthma and to several AIDs [21,22,23]. The *GSDMB* gene (also known as *GSDML*, *PP4052*, or *PRO2521*) encodes the Gasdermin B protein (416 amino acids; 47 KDa), which belongs to the gasdermin-domain containing family. This protein family consists of four members (GSDMA to D) that share several conserved residues at the N- and C-terminal regions [24]. Although *GSDMB* resulted dysregulated in a variety of cancers [25,26,27,28], the exact function of this protein is still largely unknown.

*GSDMB* is characterized by a complex pattern of AS; in the University of California, Santa Cruz (UCSC) Genome Browser database [29] four reference transcripts are annotated, characterized by the alternative skipping of exon 6, exon 7, or both. A differential expression of splicing isoforms was detected in gastrointestinal and hepatic cancers in respect to non-tumor tissues, with a correlation between an increased amount of shorter isoforms and cancer development and progression [26,30]. Moreover, in a study aimed at identifying polymorphisms associated with autoimmune/inflammatory diseases and affecting splicing, Morrison and colleagues [31] identified the rs11078928 (A > G) polymorphism, impacting on the invariant AG dinucleotide at the acceptor splice site of *GSDMB* intron 5. This polymorphism was associated with the production of an alternative transcript lacking exons 5, 6, 7, and 8 (Δ5–8; still not reported in the database), as well as with changes in the percentage of exon 6 skipping. In particular, homozygotes for the G allele showed a decreased expression of the Δ5–8 transcript and an increase of the isoform skipping exon 6.

In this work, we aimed at the in-depth characterization of *GSDMB* AS and backsplicing profiles, with a specific focus on the possible dysregulation of AS and circRNA patterns in peripheral blood mononuclear cells (PBMCs) of patients affected by MS, an autoimmune disorder already associated with alterations in AS [2,12].

## 2. Results

### 2.1. Identification of Novel GSDMB Alternative Splicing (AS) Isoforms

**Figure 1:**
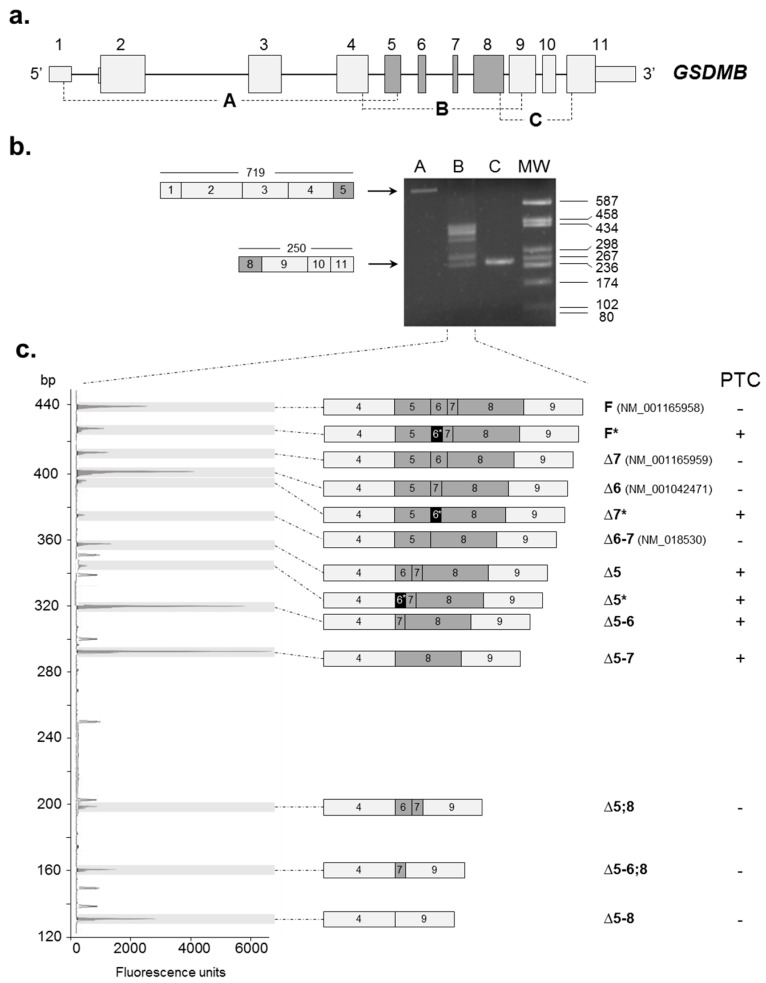
*GSDMB* alternative splicing (AS) pattern. (**a**) Schematic representation of the *GSDMB* gene. Exons are represented by boxes and are approximately drawn to scale; untranslated regions (UTRs) are depicted by smaller boxes; introns are represented by lines. Constitutive exons are colored in light grey and alternative exons in dark grey. The three reverse-transcription (RT)-PCR assays (A, B, and C) performed to cover the *GSDMB* coding region are indicated; (**b**) An illustrative agarose gel (2%) shows the amplification products of the three RT-PCR assays. For “A” and “C” assays, the unique amplification product and their length, expressed as base pairs (bp), are schematized on the left. For the “B” assay, multiple specific amplification products were obtained, corresponding to several AS events involving exons 4 to 9, which were further analyzed by both fluorescent RT-PCR and direct sequencing (see further). MW: molecular-weight marker (pUC9-*Hae*III); (**c**) The left panel represents a demonstrative GeneMapper window showing the fluorescent RT-PCR products of the “B” assay (on RNA extracted from a healthy individual, heterozygous for the rs11078928 polymorphism), using the forward primer labelled with the HEX fluorophore. The filled peaks, shaded in grey, correspond to the RT-PCR products; empty peaks represent the size standard (ROX-500 HD). The vertical axis indicates the length of amplified products in bp, whereas the horizontal axis shows fluorescence units. The schematic representation and the name of the corresponding isoform product are indicated on the right side. The alternative exon 6* is depicted in black. F”: full length, “*” indicates the presence of exon 6* in the isoform, “∆” is followed by the skipped exon, separated by “-” or “,” if the exons are contiguous or not, respectively. The accession number of University of California, Santa Cruz (UCSC) Genome Browser-annotated isoforms is also indicated. The presence/absence in the transcript of a premature termination codon (PTC) is indicated by a “+”/“−”, respectively.

To characterize the *GSDMB* AS pattern, we performed three reverse-transcription polymerase chain reaction (RT-PCR) assays on RNA extracted from PBMCs of nine healthy individuals (three males and six females). Primer couples were designed in order to obtain partially overlapping fragments (A, B, and C) covering the whole *GSDMB* coding region, thus catching most of the possible alternative transcripts (Figure 1a). The choice to analyze nine subjects—three for each genotype of the rs11078928 polymorphism—was driven by the observation that these alleles were previously associated with different *GSDMB* AS patterns [31].

Only the region spanning exons 4–9 (RT-PCR assay “B”, Figure 1b) showed multiple AS products, whereas no evident AS events were detectable in the regions comprising exons 1–5 and 8–11 (RT-PCR assays “A” and “C”, Figure 1b). To better characterize the identified AS events, we performed a fluorescent-competitive RT-PCR assay with primers mapping within exons 4 and 9. Besides confirming the presence of the already-reported splicing isoforms, here referred to as F (full length), Δ7, Δ6, Δ6–7, Δ5–8 (skipping exon 7, exon 6, exons 6 and 7, and exons 5, 6, 7, 8, respectively), we showed at least eight additional transcripts (Figure 1c). In particular, we detected five isoforms characterized by the skipping of exon 5 alone or in combination with other exons (here named as Δ5, Δ5–6, Δ5–7, Δ5;8 and Δ5–6;8). The remaining three isoforms are characterized by the inclusion of a shorter exon 6 (exon 6*; see also further) and are referred to as F*, Δ5*, and Δ7*. The identity of all of these AS transcripts was confirmed by Sanger sequencing.

The analysis of this complex AS pattern evidenced the preferential associations of specific splicing events, i.e.,: (i) the inclusion of exon 5 in the mature transcript is always associated with the presence of exon 8; and (ii) the inclusion of exon 6 (or 6*) is always accompanied by the presence of a contiguous exon (5 or 7).

### 2.2. GSDMB Is Regulated by Nonsense-Mediated mRNA Decay (NMD)

**Figure 2:**
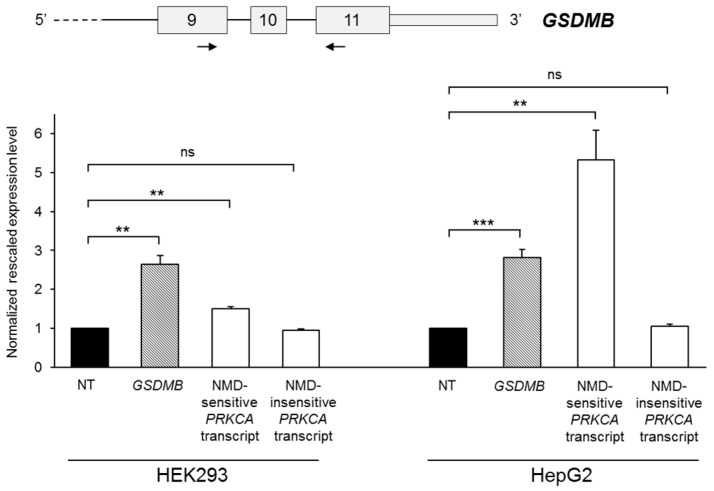
Evaluation of *GSDMB* susceptibility to nonsense-mediated mRNA decay (NMD). The upper panel shows a partial scheme of the *GSDMB* gene and the position of the primer couple used in the semi-quantitative real-time RT-PCR assay. The lower panel represents *GSDMB* total expression levels in HEK293 and HepG2 cells lines untreated (NT) or treated for 8 h with cycloheximide. Expression levels of two *PRKCA* transcripts, sensitive and insensitive to NMD, are also shown. The expression level of the untreated sample was set to 1. Bars represent means + SEM (standard error of the mean) of three independent experiments, each performed in triplicate. Significance levels of *t*-tests are shown. ** *p* < 0.01; *** *p* < 0.001; ns: not significant.

Considering that six out of eight of the newly-described *GSDMB* splicing isoforms are characterized by the presence of a premature termination codon (PTC) (Figure 1c), we tested the susceptibility of *GSDMB* to nonsense-mediated mRNA decay (NMD), a translation-coupled mechanism that degrades mRNAs containing PTCs to prevent the production of truncated proteins [32]. HEK293 and HepG2 cell lines were treated with cycloheximide, an inhibitor of the protein synthesis and, consequently, of the NMD pathway. After 8 h of treatment, total RNA was extracted and *GSDMB* expression levels were evaluated by semi-quantitative real-time RT–PCRs, using primers anchored to the 3′ terminus region of the transcript, common to all splicing variants (Figure 2). Two different transcripts of the *PRKCA* gene (respectively sensitive and insensitive to NMD) were used as positive and negative controls of the treatment [11]. We observed a significant increase of the total level of *GSDMB* after cycloheximide treatment in both cell lines with respect to the untreated sample (HEK293: 2.64-fold, *p* = 0.0019; HepG2: 2.8-fold, *p* = 0.00090) (Figure 2), suggesting that, in physiologic conditions, *GSDMB* mRNA levels are downregulated by NMD.

### 2.3. GSDMB AS Is Dysregulated in Relapsing Remitting (RR)-Multiple Sclerosis (MS) Patients

**Figure 3:**
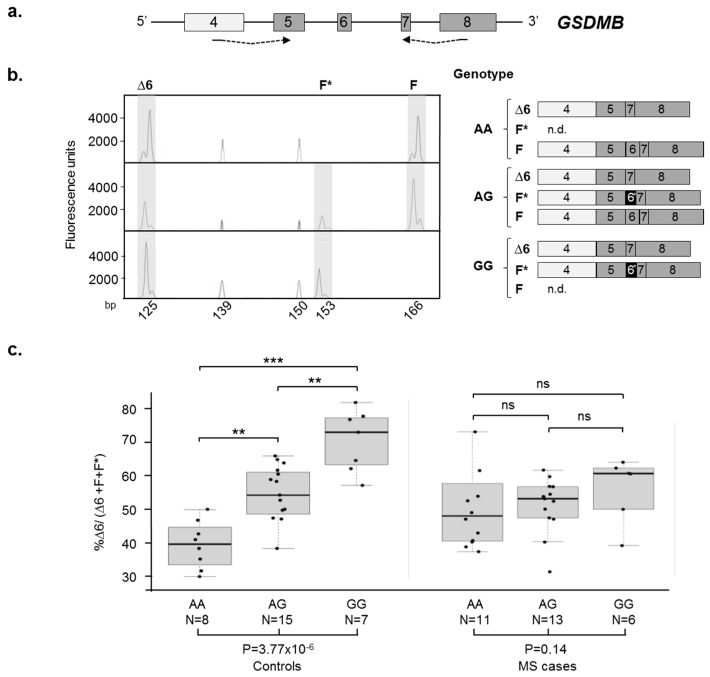
Analysis of exon 6/6* AS in multiple sclerosis (MS) cases and healthy controls. (**a**) Schematic representation of the *GSDMB* genomic region comprised between exons 4 and 8. Exons are approximately drawn to scale; alternative exons are depicted in dark grey, as in Figure 1. The dashed arrows below the scheme indicate the primer couple used in the fluorescent-competitive RT-PCR assay; the forward primer is labelled with the HEX fluorophore; (**b**) The left panel shows three GeneMapper windows, representing an example of the fluorescent products obtained for each of the rs11078928 genotypes. The peaks shaded in grey correspond to the RT-PCR products; those not shaded represent the size standard (ROX-500 HD). The schematic representation of the obtained products for each genotype is shown on the right. Exon 6* is depicted in black; (**c**) Boxplots showing the percentage of the Δ6 isoform with respect to the sum of Δ6, F*, and F isoforms measured on RNA extracted from 30 relapsing remitting (RR)-MS cases and 30 controls, grouped on the basis of the rs11078928 genotype. Boxes define the interquartile range; the thick line refers to the median. The number of subjects belonging to each group is also indicated (N). Significance levels of *t*-tests is shown above the boxplots (** *p* < 0.01; *** *p* < 0.001; ns: not significant). The one-way ANOVA *p*-values are reported below the boxplots.

Among the newly-described isoforms, F*, Δ7*, and Δ5* are characterized by the inclusion of exon 6*, a 13-nucleotides-shorter version of exon 6. Bioinformatics analyses, using the NetGene2 software, evidenced the presence of a cryptic acceptor splice site within exon 6—located 13 nucleotides downstream of the physiologic acceptor site. This cryptic site, even though showing a higher predicted score (Supplementary Figure S1), is only used in the presence of the G allele at the rs11078928 polymorphism, which inactivates the physiologic acceptor splice site of exon 6, as demonstrated by fluorescent-competitive RT-PCR assays on three control individuals with different genotypes (Figure 3a). In the AA homozygote, only the complete form of exon 6 (39 nucleotides) was detected, whereas the GG homozygote showed only the inclusion of exon 6* (26 nucleotides). The AG heterozygous individual showed both alternative forms of the exon (Figure 3b).

The fluorescent-competitive RT-PCR assay was hence applied for the quantitation of the F, Δ6, and F* isoforms on a cohort of 30 relapsing remitting (RR)-MS cases and 30 healthy controls, all genotyped for rs11078928. As expected, healthy controls showed a strict dependence of the Δ6 isoform levels upon the A/G genotype. In particular, the mean percentage of the Δ6 splicing isoform resulted in 39.7%, 53.7%, and 70.6% for the AA, AG, and GG genotypes, respectively (difference among groups *p* = 3.77 × 10^−6^). Concerning RR-MS patients, the mean percentages of Δ6 isoform were 49.1%, 51.3%, and 56.4% for AA, AG, and GG individuals, respectively, hence showing no differences among the three groups (*p* = 0.14). Therefore, contrary to what is seen in control individuals, the AS of exon 6 in RR-MS patients seems to be not dependent upon the rs11078928 genotype (difference between the distributions of cases and controls *p* = 0.00046).

Next, we decided to also evaluate in our case-control cohort the levels of the Δ5–8 isoform, which is, like Δ6, in-frame and associated with the rs11078928 polymorphism [31]. Since we observed a different amplification efficiency between the short Δ5–8 and longer *GSDMB* isoforms, we developed a digital RT-PCR assay, which is independent from PCR efficiency (Supplementary Figure S2a). Our measurements revealed that the Δ5–8 isoform accounts for a low percentage (on average 1.4%; range: 0.6% to 3.2%) of total *GSDMB* AS isoforms. As expected, in healthy controls the percentage of the Δ5–8 product was strictly related to the genotype at the polymorphism (difference among groups *p* = 1.03 × 10^−6^), with GG homozygotes expressing lower levels of the Δ5–8 isoform (Supplementary Figure S2b). Conversely, RR-MS patients showed no differences between the three groups (*p* = 0.30), revealing, also for this AS event, a loss of the dependence upon the rs11078928 genotype (difference between the distributions of cases and controls *p* = 0.018).

### 2.4. Identification of an Exonic Circular RNA (ecircRNA) Consisting of GSDMB Exons 4 and 5

**Figure 4:**
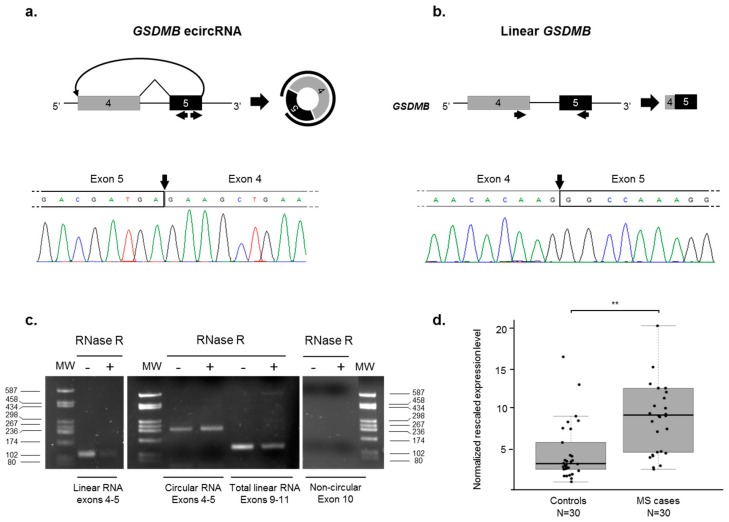
Characterization of the *GSDMB* exonic circular RNA (ecircRNA). (**a**) Schematic representation of the formation of the *GSDMB* ecircRNA through a backsplicing event between exons 4 and 5. Exons are approximately drawn to scale; exon 4 is depicted in grey, exon 5 in black. The curved arrow joins the 5′ splice site of exon 5 to 3′ splice site of exon 4. Arrows below exon 5 indicate the divergent primer couple used to detect the putative circular RNA (circRNA). On the right, a schematic representation of the circRNA is depicted, with the circular black line representing the product amplified by the primer couple. Below the scheme, direct-sequencing electropherograms show the head-to-tail splice junction, indicated by an arrow, located between *GSDMB* exons 5 and 4; (**b**) Schematic representation of the linear splicing involving exons 4–5. Arrows below exons 4–5 indicate the primer couple used in RT-PCR assays. Electropherograms showing the junction between exons 4 and 5 is also reported; (**c**) Agarose gel (2%) with the results of the RNase R treatment. The expression of the *GSDMB* ecircRNA was evaluated by RT-PCR in untreated (−) or RNase R-treated (+) RNA of a healthy control, using the divergent primer couple within exon 5. Expression of total *GSDMB* linear mRNA was also evaluated, using the RT-PCR assay shown in Figure 2 (linear product; exons 9–11), the assay reported in (**b**) (linear product; exons 4 and 5), as well as an assay using a divergent primer couple on exon 10 (negative control; no circRNA detected). MW: molecular-weight marker (pUC9-*Hae*III); (**d**) Boxplots show expression levels of the *GSDMB* ecircRNA measured by semi-quantitative real-time RT-PCR in PBMCs of 30 MS cases and 30 healthy controls. Boxes define the interquartile range; the thick line refers to the median. Results were normalized to expression levels of the *HMBS* housekeeping gene. The number of subjects belonging to each group is also indicated (N). The significance level of *t*-test analysis is shown. ** *p* < 0.01.

Considering the complexity of its AS pattern, we decided to comprehensively evaluate whether any *GSDMB* exon might be subjected to backsplicing producing ecircRNAs. To this aim, a series of RT-PCR assays were performed, with divergent primer couples tagging all exons (Supplementary Table S1), on RNA extracted from PBMCs of two healthy controls. The only detectable amplification product was obtained with primers mapping in exon 5. Direct sequencing of this putative ecircRNA revealed the presence of the backspliced exons 5 and 4, joined by a head-to-tail splice junction (Figure 4a,b). To further validate this ecircRNA, we performed RNase R treatment, known to degrade linear RNAs and to enrich the sample for resistant circRNA products [33]. RT-PCR assays performed on RNase R-treated RNA of a healthy control, and analyzed during the exponential phase of the PCR reaction, showed a differential sensitivity to the treatment of the ecircRNA compared to the linear *GSDMB* transcripts. In particular, the densitometric analysis, performed with the ImageJ software (National Institute of Health, Bethesda, MD, USA, https://imagej.nih.gov/ij/), evidenced a 1.5-fold enrichment of the *GSDMB* circular product and an 86% and 53% depletion of the *GSDMB* linear ones in the treated samples (for exons 4–5 and 9–11, respectively; Figure 4c).

Given that *GSDMB* ecircRNA is a newly described RNA species, we evaluated its expression levels in a panel of human tissues and brain regions by semi-quantitative real-time RT-PCR assays. As a comparison, we also analyzed the expression of total *GSDMB* linear transcript (Supplementary Figure S3a). In general, the level of the *GSDMB* linear mRNA was significantly higher than the one of the ecircRNA (real-time mean *C*_t_ 27.4 ± 1.9 vs. 33.1 ± 1.4, assuming equal efficiency of the two amplicons). *GSDMB* circRNA expression was detectable in almost all of the analyzed tissues, with the highest levels in colon and small intestine and the lowest in heart and kidney (Supplementary Figure S3b). The pattern of expression in the analyzed tissues of the linear and circular *GSDMB* transcripts was comparable, except for ovary, spleen, and brain (Pearson’s correlation coefficient = 0.85, *p* = 0.00080). The *GSDMB* circRNA showed also a specific expression pattern in brain regions, with very low expression levels in frontal cortex and corpus callosum and high levels in entorhinal and temporal cortex. Again, a similar expression profile was found for linear and circular *GSDMB* expression in most brain regions, with the exception of the corpus callosum and the occipital cortex (Pearson’s correlation coefficient = 0.78, *p* = 0.021) (Supplementary Figure S3c).

Considering the observed differences in AS characterizing the MS status, we decided to verify if also the expression levels of this backspliced product were different between RR-MS cases and controls. Real-time RT-PCR performed on RNA extracted from PBMCs showed a significant upregulation of the *GSDMB* ecircRNA in MS cases with respect to controls (2.8-fold, *p* = 0.0011) (Figure 4d). The levels of expression of the *GSDMB* ecircRNA were independent from the rs11078928 genotype, both in MS cases and healthy controls (data not shown).

## 3. Discussion

Despite the description of several associations of *GSDMB* polymorphisms with AIDs, the molecular mechanism/s linking the genetic variants to these diseases remained elusive [22,23]. Some light was shed by Morrison and colleagues [31], who, by using an unbiased bioinformatics approach, identified several polymorphisms associated with AID susceptibility showing a potential effect on splicing of different candidate genes. Among the identified variants, the rs11078928 polymorphism in the *GSDMB* gene was related both to a decreased inclusion of exon 6 in the mature transcript, and to a reduced level of the potentially-deleterious Δ5–8 isoform [31]. With this background, we decided to comprehensively describe the *GSDMB* AS and backsplicing patterns, with a particular focus on MS, an inflammatory, demyelinating AID of the central nervous system [34].

Concerning linear splicing, we used fluorescent-competitive RT-PCR, which is very convenient to investigate AS isoforms, since it can detect multiple isoforms in the same assay. This approach can overcome the disadvantages of using isoform-specific TaqMan assays and allowed us to identify eight previously-undetected in-frame and out-of-frame alternative transcripts of the gene (Figure 1). The specific measurements of the in-frame Δ6 and Δ5–8 isoforms evidenced, as already reported [31], a clear dependence of their levels upon the rs11078928 genotype, at least in controls (Figure 3 and Supplementary Figure S2). Conversely, a significant dysregulation of *GSDMB* AS profile was evident in RR-MS cases, whose levels of exon 6 and exons 5–8 skippings were unrelated to the rs11078928 polymorphism. This same dysregulation of AS profiles in MS cases, characterized by the loss of genotype-dependent splicing in the presence of a polymorphism directly affecting a splice site, has been already described for at least two other genes (*PRKCA* and *NFAT5*) [11,12], thus possibly representing a specific “signature” of the disease status. The loss of genotype-dependent regulatory mechanisms might be explained by the extensive general dysregulation of proteins involved in the splicing process observed by us and other groups in MS [12,35,36,37]. In fact, it is well known that—besides *cis*-acting variants—differences in the levels of AS isoforms may be due to alterations in *trans*-acting factor levels [38]. Interestingly, variations in the expression of *trans*-acting factors, such as SR proteins and hnRNPs, were found also in cancer [39,40], in which a differential expression of *GSDMB* splicing isoforms was repeatedly evidenced [26,28,30]. Moreover, besides alterations in genotype-dependent AS, we also measured a significant downregulation in MS patients of the overall amount of *GSDMB* mRNA by digital RT-PCR on constitutively expressed exons (9–11) (see Supplementary Figure S4).

Biogenesis of circRNAs has been recently proposed among mechanisms of AS regulation, due to the use of the canonical spliceosomal machinery and to the competition with linear splicing for splice sites [17,41]. Given the complex architecture of the *GSDMB* AS pattern, we hypothesized a similarly complex backsplicing pattern, and hence designed a set of assays covering the whole *GSDMB* coding region, in order to catch all possible backsplicing events. We detected and validated only one ecircRNA, including exons 5 and 4, which is annotated both in the circBase (accession number hsa_circ_0106803) and CIRCpedia (accession number HSA_CIRCpedia_78516) databases as expressed in different brain regions [42,43]. Exon 4 is not normally skipped from the linear transcript; however, this is not surprising, as exon skipping may be not necessary for ecircRNA formation [44]. We found this ecircRNA in all tissues and brain regions expressing linear *GSDMB*; our experimental results did show a positive correlation between linear and circRNA *GSDMB* expression levels, with the exception of some tissues, a behavior that has already been observed in several studies [45,46,47]. Intriguingly, the evaluation of *GSDMB* ecircRNA expression in our cohort of MS cases and controls evidenced a 2.8-fold upregulation in RR-MS patients’ PBMCs. Considering the high stability of circRNAs, due to their resistance to exonucleases normally involved in linear transcript degradation and to their high expression in peripheral whole blood [48,49], our ecircRNA may hence represent a potential biomarker for MS. Indeed, the clinical usefulness of these molecules has been already discussed for cancer and neurological diseases [50,51]. On a global transcriptome perspective, given that a general alteration of splicing seems to be a feature of MS [12,35,36,37], it would be interesting to verify whether a concomitant widespread alteration of backsplicing is also present in MS patients.

CircRNA biogenesis can compete with pre-mRNA splicing, correlating with the levels of linear mRNAs skipping the circularized exons [17,18]. Concerning *GSDMB*, however, we could not detect the presence of exon 4–5 skipped transcripts in tissues expressing our ecircRNA (data not shown). This could be either due to the fact that exon 5 to 4 backsplicing is mutually exclusive with the corresponding linear splicing, or to the fact that exon 4–5 skipped mRNAs are generated but rapidly degraded by NMD.

It remains difficult to establish whether and how alterations in circRNA production and *GSDMB* AS may contribute to the susceptibility to MS. On one hand, the possible functional roles of the circRNA are still needing to be explored. For example, among the six miRNAs having more than one target site in the circRNA (as predicted by the PITA algorithm, https://genie.weizmann.ac.il/pubs/mir07/index.html), at least two (miR-1275 and miR-149) were previously found to be differentially expressed in blood in MS patients compared to controls [52,53]. On the other hand, current knowledge concerning the protein function is limited. Recently, another member of the gasdermin family, GSDMD, was demonstrated to mediate pyroptosis, an inflammasome-dependent cell death, resulting in pore formation in the plasma membrane and in release of inflammatory contents [54]. This mechanism is triggered by the GSDMD N-domain, which is released through a cleavage by inflammatory caspases [55,56]. Moreover, also the N-domains of both the GSDMB protein and other gasdermin family members are able to induce pyroptosis in HEK293T cells [57]. Interestingly, altered signaling of inflammasome may be involved in several AIDs, including MS, where higher levels of inflammasome-related genes were found [58,59,60].

Further sustaining a possible role of *GSDMB* in the pathogenesis of MS, the knockdown of *GSDMB* in memory CD4^+^ T-cells was shown to augment the production of cytokines such as tumor necrosis factor (TNF), interleukin (IL)-13, and IL-16 [61]. In addition, Das and colleagues [62] demonstrated that the *GSDMB* Δ6 isoform overexpression in primary human bronchial epithelial cells can lead to increased expression levels, among others, of the transforming growth factor beta 1 (*TGFB1*) and matrix metallopeptidase 9 (*MMP9*) genes. *TGFB1* expression levels were shown to be decreased in MS leukocytes [63] and to be increased in MS-patient serum after interferon beta-1b (IFN-β1b) therapy [64]. Concerning *MMP9*, it is considered a marker of disease activity in MS, due to its upregulation in patient serum and cerebrospinal fluid [65].

## 4. Materials and Methods

### 4.1. Subjects

All analyzed patients were diagnosed with RR-MS, the most common form of MS [66]. At the time of blood withdrawal, all cases (7 males, 23 females) were in the remission phase and had not received any immunomodulatory treatment for at least a month. The age- and sex-matched healthy controls declared no familial history for autoimmune or neurodegenerative diseases.

This study was approved by local Ethical Committees (protocol 576CE, study CE38/05, 15 July 2005) and was conducted according to the Declaration of Helsinki and to the Italian legislation on sensible data recording. All the participants in the study signed an informed consent.

### 4.2. DNA and RNA Samples

DNA samples were extracted from peripheral blood using an automated DNA extractor (Maxwell 16 System; Promega, Madison, WI, USA). PBMCs were isolated by means of centrifugation on a Lympholyte Cell separation medium (Cederlane Laboratories Limited, Hornby, ON, Canada) gradient. RNA extraction was performed using the EuroGold Trifast kit (Euroclone, Wetherby, UK).

*GSDMB* linear and ecircRNA expression levels were measured by using RNA from a panel of 10 human tissues (Thermo Fisher Scientific, Waltham, MA, USA) and eight human brain regions (Takara Bio USA, Mountain View, CA, USA).

DNA and RNA concentrations were measured using the NanoDrop 2000 Spectrophotometer (Thermo Fisher Scientific).

### 4.3. RT-PCR, Fluorescent-Competitive RT-PCR, and Semi-Quantitative Real-Time RT-PCR

Random hexamers (Promega) and the Superscript-III Reverse Transcriptase (Thermo Fisher Scientific) were employed to perform first-strand cDNA synthesis, according to the manufacturer’s instructions. One microliter of the RT reaction was used as template for the subsequent RT-PCR, fluorescent-competitive RT-PCR, or semi-quantitative real-time RT-PCR assays.

To characterize global *GSDMB* AS and backsplicing patterns, RT-PCR reactions were performed under standard conditions on a Mastercycler EPgradient (Eppendorf, Hamburg, Germany) using the GoTaq DNA Polymerase (Promega).

To describe *GSDMB* AS in the region including exons 4–9 and to quantitate the Δ6 isoform, fluorescent-competitive RT-PCR assays were performed using a HEX-labeled primer. Amplification products were separated by capillary electrophoresis on an ABI-3500 Genetic Analyzer (Thermo Fisher Scientific) and the peak areas were measured by the GeneMapper v4.0 software (Thermo Fisher Scientific). The percentage of each specific isoform was measured by calculating the ratio of the relevant peak over the sum of all of the fluorescence peak areas (set as 100%).

Semi-quantitative real-time RT-PCRs were accomplished by using the FastStart SYBR Green Master mix (Roche, Basel, Switzerland) and a touchdown thermal protocol on a LightCycler 480 (Roche). *GJA1* (Gap Junction Protein α 1, expressed in HEK293), *GJB1* (Gap Junction Protein β 1, expressed in HepG2), or *HMBS* (hydroxymethylbilane synthase) expression levels were used as housekeeping genes for NMD-susceptibility assays and for linear and circular *GSDMB* quantitation, respectively. Reactions were performed at least in triplicate, and expression data were analyzed using the GeNorm software [67].

All primer couples used in these reactions are reported in Supplementary Table S1.

### 4.4. Digital RT-PCR

To measure *GSDMB* Δ5–8 isoform levels and total *GSDMB* transcript, digital RT-PCR reactions were performed on a QuantStudio 3D Digital PCR System (Thermo Fisher Scientific) using the QuantStudio 3D Digital PCR Master Mix (Thermo Fisher Scientific) and 1 μL of cDNA as template. Custom TaqMan assays were designed to amplify *GSDMB* Δ5–8 isoform and total *GSDMB* isoforms. The sequences of primers and probes used in digital RT-PCR assays are listed in Supplementary Table S1.

Each reaction mixture was loaded onto a QuantStudio 3D Digital PCR Chip (Thermo Fisher Scientific) and cycled for 40 cycles using standard conditions. End-point fluorescence data were analyzed through the QuantStudio 3D Digital PCR Instrument and the QuantStudio 3D Analysis Suite (Thermo Fisher Scientific), according to the manufacturer’s instructions.

### 4.5. Direct Sequencing

To genotype the rs11078928 polymorphism and to confirm the identity of the diverse *GSDMB* isoforms and of the identified ecircRNA, direct sequencing of relevant PCR or RT-PCR products was performed. For genotyping experiments, the genomic region containing the variant was amplified by standard PCR reactions performed on 20 ng of genomic DNA by using the GoTaq DNA polymerase (Promega). The specific primer couples used in these reactions are listed in Supplementary Table S1.

Sequencing reactions were prepared with the BigDye Terminator Cycle Sequencing Ready Reaction Kit v1.1 (Thermo Fisher Scientific) and run on an ABI-3500 Genetic Analyzer (Thermo Fisher Scientific).

### 4.6. Cell Cultures and Sensitivity to NMD

HEK293 cells were cultured in Dulbecco modified Eagle’s medium (EuroClone), HepG2 cells in RPMI 1640 (EuroClone) with the addition of sodium pyruvate (1 mM; Sigma-Aldrich, Saint Louis, MO, USA). In both media, 10% fetal bovine serum, 1% glutamine, and antibiotics (100 U/mL penicillin and 100 μg/mL streptomycin; EuroClone) were added. Cells were grown at 37 °C in a humidified atmosphere of 5% CO_2_ and 95% air, according to standard procedures.

Both cell lines were plated at a density of 5.5 × 10^6^ per 10-cm dish and, after 72 h, treated with 100 μg/mL cycloheximide (Sigma-Aldrich), an inhibitor of protein synthesis. Untreated samples were incubated with the drug solvent (dimethyl sulfoxide). Total RNA extraction was performed after 8 h of treatment, and *GSDMB* expression levels were measured by semi-quantitative real-time RT-PCR, as previously described.

### 4.7. RNase R Treatment

Two micrograms of RNA extracted from PBMCs of a healthy control were digested with six units of RNase R (Epicentre, Madison, WI, USA) for 10 min at 37 °C, according to manufacturer’s instructions. The treated RNA was purified by phenol-chloroform extraction. Five hundred nanograms of the treated RNA and an equal amount of untreated sample were used for standard RT reactions.

### 4.8. Statistical Analyses

*t*-Test, ANOVA, and correlation analyses were performed using the R software [68]. Correlation between *GSDMB* linear and ecircRNA expression profiles was calculated using the Pearson’s correlation. Pearson’s coefficients <−0.5 and >0.5 were considered as anti-correlation and positive correlation, respectively. *p*-Values < 0.05 were considered statistically significant.

## 5. Conclusions

Literature data suggest an involvement of *GSDMB* in inflammation and autoimmunity. Our results fit well with this hypothesis, and specifically provide evidence of alterations of *GSDMB* AS and backsplicing profiles in MS, highlighting a possible involvement of *GSDMB* isoform unbalance in the disease pathogenesis.

## Supplementary Materials

Supplementary materials can be found at www.mdpi.com/1422-0067/18/3/576/s1.

## Abbreviations

AID	Autoimmune disease
AS	Alternative splicing
MS	Multiple sclerosis
hnRNP	Heterogeneous nuclear ribonucleoprotein
circRNA	Circular RNA
ecircRNA	Exonic circular RNA
*GSDMB*	Gasdermin B gene
*GSDML*	Alternative name for Gasdermin B gene
*PP4052*	Alternative name for Gasdermin B gene
*PR02521*	Alternative name for Gasdermin B gene
*GSDMA-D*	Gasdermin A to D genes
UCSC	University of California, Santa Cruz
Δ#	Transcript lacking exon number#
PBMC	Peripheral blood mononuclear cell
RT-PCR	Reverse transcription-polymerase chain reaction
UTR	Untranslated region
F	*GSDMB* full-length transcript
6*	Alternative isoform of *GSDMB* exon 6
PTC	Premature termination codon
NMD	Nonsense-mediated mRNA decay
NT	Not treated
*PRKCA*	Protein kinase C alpha gene
*HMBS*	Hydroxymethylbilane synthase gene
SEM	Standard error of the mean
ns	Not significant
RR	Relapsing remitting
ANOVA	Analysis of variance
*NFAT5*	Nuclear factor of activated T-cells 5 gene
SR proteins	Serine-arginine proteins
TNF	Tumor necrosis factor
IL-13	Interleukin 13
IL-16	Interleukin 16
*TGFB1*	Transforming Growth Factor Beta 1 gene
*MMP9*	Matrix Metallopeptidase 9 gene
IFN-β1b	Interferon beta-1b
*GJA1*	Gap junction alpha-1 protein gene, also known as connexin 43
*GJB1*	Gap Junction Beta 1 protein gene, also known as connexin 32
