# Supplementary material for: The Characterization of GSDMB Splicing and Backsplicing Profiles Identifies Novel Isoforms and a Circular RNA That Are Dysregulated in Multiple Sclerosis
Source: Int J Mol Sci. 2017 Mar 7;18(3):576. doi: 10.3390/ijms18030576 (PMC5372592; doi:10.3390/ijms18030576)
Supplement: Supplementary file 1 [file ijms-18-00576-s001.pdf]

Supplementary Materials

Article

The Characterization of *GSDMB* Splicing and Backsplicing Profiles Identifies Novel Isoforms and a Circular RNA that are Dysregulated in Multiple Sclerosis

Giulia Cardamone, Elvezia Maria Paraboschi, Valeria Rimoldi, Stefano Duga, Giulia Soldà and Rosanna Asselta

List of contents:

- Supplementary Figure 1
- Supplementary Figure 2
- Supplementary Figure 3
- Supplementary Figure 4
- Supplementary Table 1

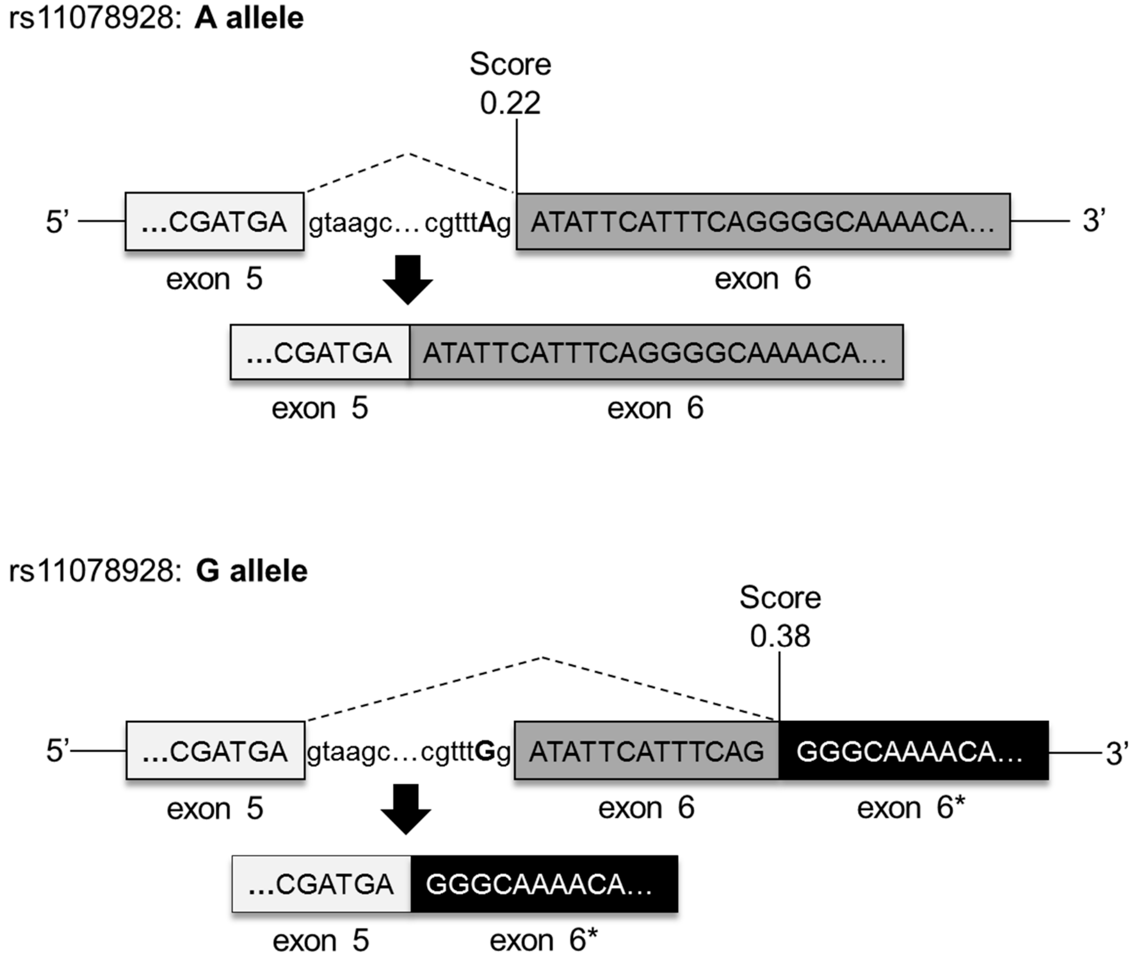

Figure S1: Bioinformatics analysis of splicing site prediction. Bioinformatics analyses were performed on splice acceptor site of intron 5 using the NetGene2 software (http://www.cbs.dtu.dk/services/NetGene2/). Exons and introns, both not to scale, are represented by boxes and lines, respectively. Dotted lines represent the predicted splice event, for which the assigned score is reported (values range from 0 to 1). The partial sequences of exons 5 and 6 (in uppercase) and of introns 5 (lowercase) are also reported. The upper scheme is characterized by the presence of A at the level of the rs11078928 polymorphism, whereas the lower scheme corresponds to the presence of the minor allele G. In this last case, exon 6 is represented in grey (excluded portion) and in black (retained portion, corresponding to exon 6*).

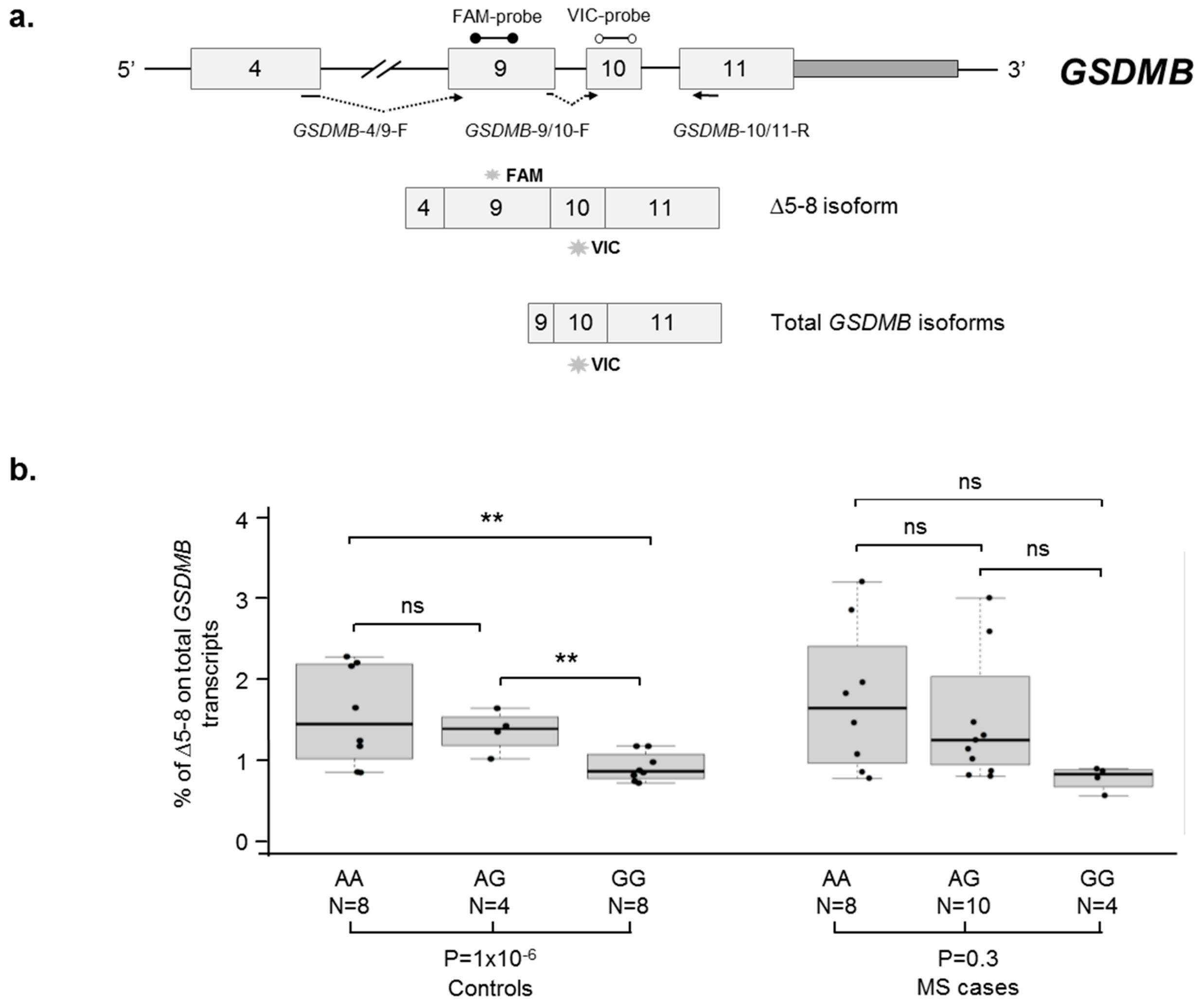

Figure S2: Quantitation of Δ5–8 isoform levels in MS cases and controls by digital RT-PCR. (**a**) Schematic representation of the digital RT-PCR assay. Upper panel: partial scheme of *GSDMB* gene, showing the primer couples and the TaqMan probes used in the assay. Primers are represented by arrows; TaqMan probes are represented by lines with dots, indicating the reporter and the quencher dyes. Lower panels: representation of the possible products amplified by the assay. The short Δ5–8 isoform is detected by the fluorescent signal derived by both FAM and VIC reporter dyes; the remaining *GSDMB* isoforms are detected by the fluorescent signal only derived by the VIC reporter dye. (**b**) Distribution of the absolute quantity of the Δ5–8 isoform (stratified upon the rs11078928 genotype) in MS cases and controls. Percentages of the Δ5–8 isoform respect to *GSDMB* total transcript are shown. Boxes define the interquartile range; the thick line refers to the median. The number of subjects in which the assays was performed is also indicated. Significance levels of t-tests is shown above the boxplots (** $P < 0.01$; ns: not significant). The one-way ANOVA P values are reported below the boxplots.

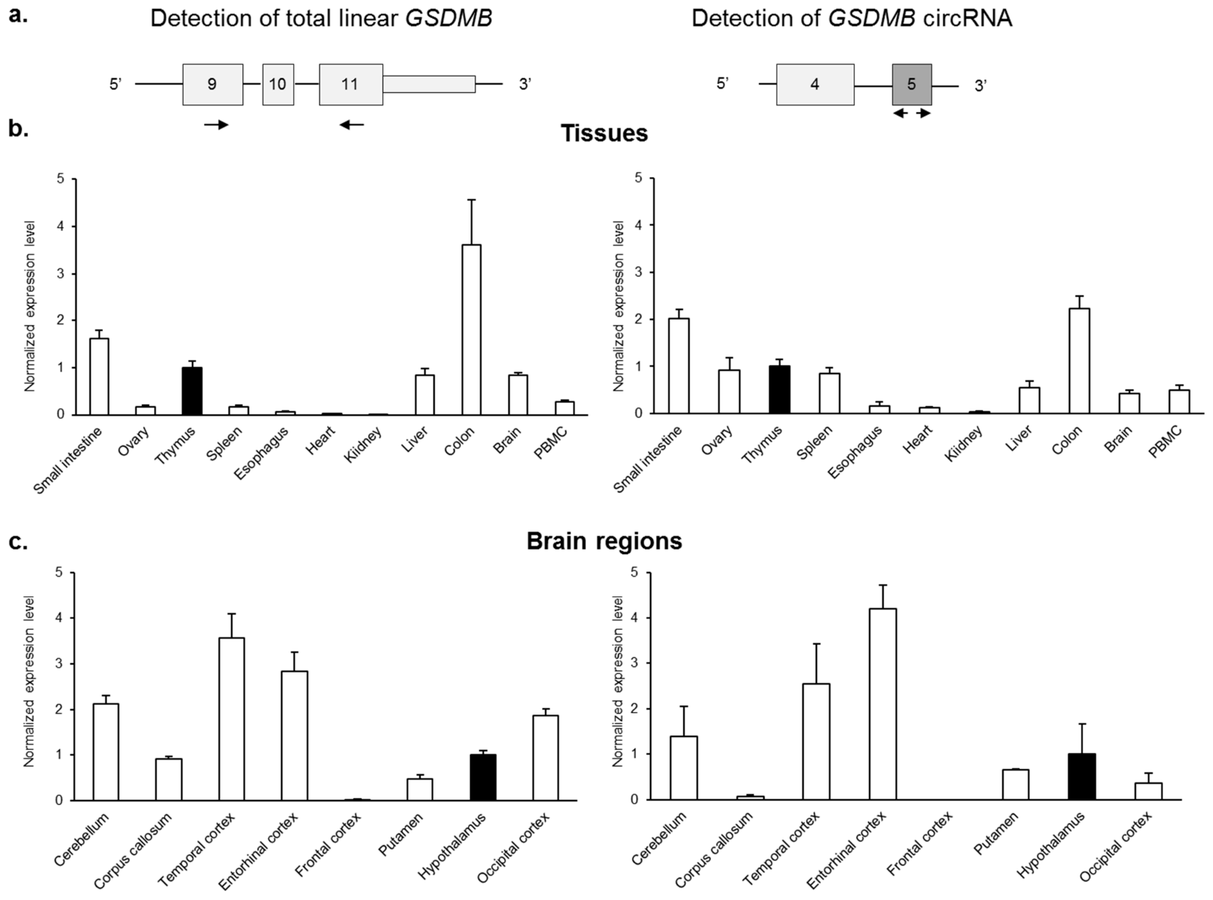

Figure S3: Linear and circular *GSDMB* expression levels in human tissues and brain regions. (**a**) Schematic representation of the real-time RT-PCR assays performed to detect the expression levels of linear *GSDMB* and of the circRNA consisting of *GSDMB* exon 4 and 5. Exons are represented by boxes and are drawn to scale; introns by lines. The primer couples used are also indicated (arrows below the scheme). (**b**) Expression levels of linear *GSDMB* (on the left) and the ecircRNA (on the right) were analyzed in a commercial panel of human tissues (each comprising RNA derived from at least three donors) and in PBMCs from two healthy individuals. Expression analyses were performed by semi-quantitative real-time RT-PCR; results are normalized to *HMBS* expression levels and are rescaled setting 1 as the values of the thymus (indicated in black). (**c**) Expression levels of linear *GSDMB* (on the left) and the ecircRNA (on the right) analyzed in a commercial panel of brain regions (each comprising RNA derived from one or more donors). In this case, results are rescaled setting 1 as the values of the hypothalamus (indicated in black).

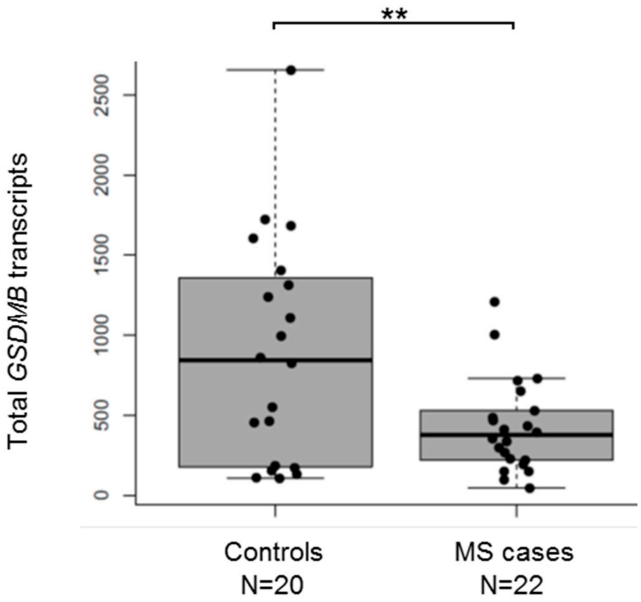

Figure S4: Absolute quantitation of total *GSDMB* levels in MS cases and controls by digital RT-PCR. The primer couple and probe used to quantitate GSDMB are shown in Supplementary Figure 2a (exons 9–11). Boxes define the interquartile range; the thick line refers to the median. The number of subjects in which the assays was performed is also indicated. Significance level of t-test is shown above the boxplots (** P < 0.01).

Table S1: Primer couples used for all the assays.

Primer	*Sequence (5′-3′)*	*Localization*	*Application*
GSDMB-1-F<br>*GSDMB*-5-R	GGGGATTCTCACAACTTCCA<br>CTCCTTGTTGGGGAAGACAA	Exon 1<br>Exon 5	Detection of AS isoforms by competitive RT-PCR
GSDMB-4-F<br>*GSDMB*-9-R	[HEX]GATCTCTCAGGGCCATCTCA<br>CTTCTACCAAGACCCCAGCA	Exon 4<br>Exon 9	Detection of AS isoforms by fluorescent-competitive RT-PCR$^{\Delta}$
GSDMB-8-F<br>*GSDMB*-11-R	GGCAGGATCTAGAGCAAAGA<br>TGCTCCATGACAGATTTCAC	Exon 8<br>Exon 11	Detection of AS isoforms by competitive RT-PCR
rs11078928-F<br>rs11078928-R	AGGCAGGAGAATTGCTTGAA<br>GGTGCGTCTTACCACATCCT	Intron 5<br>Intron 6	Genotyping of rs11078928
GSDMB-9-F<br>*GSDMB*-11-R	TGCAAAAGCCATTCTGGACT<br>TGCTCCATGACAGATTTCAC	Exon 9<br>Exon 11	Detection of all isoforms by semi-quantitative real-time RT-PCR†
PRKCA-3*-F<br>*PRKCA*-4/5-R	TCCCCTGTATTGCTAGTCTGC<br>TGAACTTGTGCTTGCTCCTG	Exon 3*<br>Exon 4/5 junction	Detection of a NMD-sensitive transcript by semi-quantitative real-time RT-PCR
PRKCA-3/4-F<br>*PRKCA*-4/5-R	GGACCCGACACTGATGACC<br>TGAACTTGTGCTTGCTCCTG	Exon 3/4 junction<br>Exon 4/5 junction	Detection of a NMD-insensitive transcript by semi-quantitative real-time RT-PCR
GSDMB-4/5-F-HEX<br>*GSDMB*-7/8-R	[HEX]CAGCTATAAACACAAGGGCCA<br>CCTAAACAGGATGAAGCACCA	Exon 4/5 junction<br>Exon 7/8 junction	Detection of Δ6 isoform by fluorescent-competitive RT-PCR
GSDMB-4/9-F<br>*GSDMB*-10/11-R	CCATCTCAGCTATAAACACAAGGTATC<br>TGACAGATTTCACCTGGTCCT	Exon 4/9 junction<br>Exon 10/11 junction	Detection of Δ5–8 isoform by digital RT-PCR
GSDMB-9/10-F<br>*GSDMB*-10/11-R	CCTGGATGCCCTGCTAGA<br>TGACAGATTTCACCTGGTCCT	Exon 9/10 junction<br>Exon 10/11 junction	Detection of all isoforms by digital RT-PCR
GSDMB-9-FAM	[FAM]CGCTTCTACCAAGACCCCAGCAGC[BHQ1]	Exon 9	TaqMan probe for digital RT-PCR
GSDMB-10-VIC	[VIC]TGTCTGAAGAGCAGCAGTTTGTGGCT[TAMRA]	Exon 10	TaqMan probe for digital RT-PCR
GSDMB-1-F<br>*GSDMB*-1-R	GGGGATTCTCACAACTTCCA<br>CAGTTCCTGGCCTCTGAATC	Exon 1<br>Exon 1	Detection of backsplicing products by RT-PCR
GSDMB-2-F<br>*GSDMB*-2-R	GGACACAGATGGGGACAAGT<br>CAAGGCTTCTAACGGCAATC	Exon 2<br>Exon 2	Detection of backsplicing products by RT-PCR
GSDMB-3-F<br>*GSDMB*-3-R	CCGGATATCCCAGCAGTATCT<br>TGGAAACTGCCTGAAATTGTT	Exon 3<br>Exon 3	Detection of backsplicing products by RT-PCR
GSDMB-4-F<br>*GSDMB*-4-R	GATCTCTCAGGGCCATCTCA<br>TATATTGCCGGTCGCTTTTC	Exon 4<br>Exon 4	Detection of backsplicing products by RT-PCR

<u>*GSDMB*-5-F</u><br><u>*GSDMB*-5-R2</u>	TTGTCTTCCCCAACAAGGAG<br>ATAGCTCAGGACCCGATTTG	Exon 5<br>Exon 5	Detection of backsplicing products by RT-PCR and semi-quantitative real-time RT-PCR
GSDMB-6-F<br><u>*GSDMB*-5-R2</u>	GCAAAACAAAATCCTTTCCAGAA<br>ATAGCTCAGGACCCGATTTG	Exon 6<br>Exon 5	Detection of backsplicing products by RT-PCR
GSDMB-7-F<br>*GSDMB*-6-R	GGATGGTGCTTCATCCTGTT<br>TTCTGGAAAGGATTTTGTTTTGC	Exon 7<br>Exon 6	Detection of backsplicing products by RT-PCR
<u>*GSDMB*-8-F</u><br>*GSDMB*-8-R	GGCAGGATCTAGAGCAAAGA<br>CTCCTCTGTCAGGTCCTTGAG	Exon 8<br>Exon 8	Detection of backsplicing products by RT-PCR
<u>*GSDMB*-9-F</u><br><u>*GSDMB*-9-R</u>	TGCAAAAGCCATTCTGGACT<br>CTTGTCTGGGTCCTCCATGT	Exon 9<br>Exon 9	Detection of backsplicing products by RT-PCR
GSDMB-10-F<br>*GSDMB*-10-R	TTCCTCTGTTGAAGGACCAG<br>CCTCAGCCACAAACTGCTG	Exon 10<br>Exon 10	Detection of backsplicing products by RT-PCR
GSDMB-11-F<br><u>*GSDMB*-11-R</u>	ATCGCCACTACCATCCTGTC<br>TGCTCCATGACAGATTTCAC	Exon 11<br>Exon 11	Detection of backsplicing products by RT-PCR
<u>*GSDMB*-5-F</u><br><u>*GSDMB*-4-R</u>	TTGTCTTCCCCAACAAGGAG<br>TATATTGCCGGTCGCTTTTC	Exon 5<br>Exon 4	Validation of the ecircRNA by direct sequencing
GJA1-1-F<br>*GJA1*-2-R	AAAGTACCAAACAGCAGCGG<br>CTCCAGCAGTTGAGTAGGCT	Exon 1<br>Exon 2	Reference transcript used for NMD assays in semi-quantitative real-time RT-PCR
GJB1-1-F<br>*GJB1*-2-R	GCAGCAGCAGCCAGGTGTGG<br>ATACTCGGCCAATGGCAGTA	Exon 1<br>Exon 2	Reference transcript used for NMD assays in semi-quantitative real-time RT-PCR
HMBS-F<br>*HMBS*-R	GTTCAGGAGTATTCGGGGAAACC<br>TTCCTCAGGGTGCAGGATCTG	Exon 8/9 junction<br>Exon 9/10 junction	Reference transcript used for semi-quantitative real-time RT-PCR

Underlined primers are used in multiple assays.

GSDMB, gasdermin B; *PRKCA*, protein kinase C alpha; *GJA1*, gap junction protein alpha 1; *GJB1*, gap junction protein beta 1; *HMBS*, hydroxymethylbilane synthase.

Δ This primer couple was used both in fluorescent-competitive RT-PCR assays (the primer forward is labelled with the fluorophore HEX) and in standard competitive RT-PCR assays.
